# Prof. Jas. B. McCaw, M. D.

**Published:** 1858-10

**Authors:** 


### PROF. JAS. B. McCAW, M.D.

We are very much gratified to be able to record the fact, that our friend, Dr. J. B. McCaw, the able Editor of the Virginia Medical Journal, at Richmond, Va., has been appointed to the Chair of Chemistry and Pharmacy in the Virginia Medical College.

We have no hesitation in saying, that he will be a valuable addition to the Faculty of that Institution; and that he will discharge the duties of the position with marked ability.

We greatly desire to see the advantages of the Virginia Medical College appreciated; and hope that, ere long, the Medical Department of the University may be merged in this Institution, which we are satisfied would be greatly to the advantage of both.

